# Statin Intolerance: an Overview of US and International Guidance

**DOI:** 10.1007/s11883-023-01124-z

**Published:** 2023-07-06

**Authors:** Mary Katherine Cheeley, Katarina Clegg, Connor Lockridge, Tyler J. Schubert, Laney K. Jones

**Affiliations:** 1 Grady Health System, Atlanta, GA 30303 USA; 2 Geisinger Commonwealth School of Medicine, Scranton, PA 18510 USA; 3 Department of Genomic Medicine, Geisinger, Danville, PA 17822 USA; 4 Heart and Vascular Institute, Geisinger, Danville, PA 17822 USA

**Keywords:** Statin intolerance, Adherence, Persistence, Cholesterol, Non-statin, Statin

## Abstract

**Purpose of Review:**

To review recent international and domestic definitions, considerations, and treatment algorithms for statin intolerance, and specifically, statin-associated muscle symptoms (SAMS).

**Recent Findings:**

Multiple organizations around the world have produced guidance documents to aid clinicians on managing statin intolerance. A common theme resides among all the guidance documents that most patients can tolerate statins. For those patients who cannot, healthcare teams need to evaluate, rechallenge, educate, and ensure adequate reduction of atherogenic lipoproteins.

**Summary:**

Statin therapy remains the cornerstone of lipid-lowering therapies to reduce atherosclerotic cardiovascular disease (ASCVD) and reduce mortality and morbidity. The common theme throughout all these guidance documents is the importance of statin therapy to reduce ASCVD and continual adherence to treatment. Because adverse events occur and inhibit patients from achieving adequate lowering of their atherogenic lipoproteins, trial and rechallenge of statin therapy, as well as addition of non-statin therapies, especially in high-risk patients, is also undisputed. The main differences stem from laboratory monitoring and the classification of the severity of the adverse effect. Future research should focus on consistently diagnosing SAMS so that these patients can be easily identified in the electronic health records.

## Introduction

Reducing levels of atherogenic lipoproteins has been shown to decrease risk of clinical atherosclerotic cardiovascular disease (ASCVD). Statin therapy remains the cornerstone of lipid-lowering therapy and is generally well tolerated; however, occasionally patients experience adverse effects, often called statin-associated muscle symptoms (SAMS). These symptoms include, but are not limited to, muscle pain, weakness, cramps, and fatigue. The incidence of these symptoms has been reported to be as high as 5-30% in the literature [1–3]. In clinical trials, the incidence of patient reported muscle-related complications has been reported to be as low as 3% [4•, 5•, 6, 7•, 8•, 9••]. The discrepancy may be explained by the “nocebo” effect, in which the expectation of harm results in perceived side effects that may be unrelated to the pharmacological effects of the drug [8•]. There is wide variation in the incidence of statin intolerance but often these patients can still be managed with some dose of statin therapy. Complete statin intolerance, or inability to take any dose of statin therapy, is uncommon and affects less than 5% of patients [8•]. The benefits of statin therapy greatly outweigh the potential adverse effects and so a concerted effort by the healthcare team should be made to ensure individuals at risk of ASCVD continue statin therapy. Statin intolerance guidance documents have been created to assist with re-initiating statin therapy in individuals reporting statin intolerance, which include suggestions such as giving lower or intermittent doses, among other strategies.

This review provides an overview of statin intolerance guidance documents from Latin America, Europe, Canada, and the USA. Table 1 provides the definitions on statin intolerance from each guidance.

**Table 1:** Definitions of statin intolerance

Country	Guidance	Year of publication	Definition
Latin America	Statin-associated muscle symptoms: position paper from the Luso-Latin American Consortium [4•]	2017	“Inability to tolerate at least two statins at any dose; or the inability to tolerate dose increases above rosuvastatin 5 mg, atorvastatin 10 mg, simvastatin 20 mg, pravastatin 20 mg, lovastatin 20 mg, pitavastatin 2 mg, or fluvastatin 40 mg; and symptoms or creatinine kinase (CK) changes not attributable to established drug–drug interactions and recognized conditions increasing the risk of statin intolerance.” AND “intolerance must be accompanied by intolerable muscle symptoms; or severe myopathy (muscle symptoms with CK above 7x the upper limit of normal [ULN]); and whose cause may be attributed to the statin for presenting plausible time relationship (0–12 weeks) with the introduction of statin, dose increase or introduction of a drug competing for the same metabolic pathway; and/or resolution or improvement of symptoms after discontinuation of statin; and with worsening in less than 4 weeks after the new exposure (rechallenge)”
Europe	Statin-associated muscle symptoms: impact on statin therapy-European Atherosclerosis Society Consensus Panel Statement on Assessment, Aetiology, and Management [5•]	2015	
	Update of SAMS Consensus - European Atherosclerosis Society [6]	2017	“Assessment of the probability of SAMS (statin-associated muscle symptoms) being due to a statin should take into account the nature of the muscle symptoms, the elevation in CK levels and their temporal association with statin initiation, discontinuation, and re-challenge. Note that this is a clinical definition, which may not be appropriate for regulatory purposes.”
Canada	Diagnosis, Prevention, and Management of Statin Adverse Effects and Intolerance: Canadian Consensus Working Group Update [7•]	2016	“A clinical syndrome, not caused by drug interactions or risk factors for untreated intolerance and characterized by significant symptoms and/or biomarker abnormalities that prevent the long-term use and adherence to statins documented by challenges/dechallenge/re-challenge where appropriate using at least two statins, including atorvastatin and rosuvastatin, and that leads to failure of maintenance of therapeutic goals as defined by national guidelines”
USA	NLA scientific statement on statin intolerance: a new definition and key considerations for ASCVD risk reduction in the statin intolerant patient [8•]	2022	“One or more adverse effects associated with statin therapy, which resolves or improves with dose re- duction or discontinuation, and can be classified as complete inability to tolerate any dose of a statin or partial intolerance, with inability to tolerate the dose necessary to achieve the patient-specific therapeutic objective. To classify a patient as having statin intolerance, a minimum of two statins should have been attempted, including at least one at the lowest approved daily dosage.”
	Assessment and management of statin-associated muscle symptoms (SAMS): a clinical perspective from the National Lipid Association [9••]	2022	“Muscle symptoms occurring during statin treatment without regard to causality. This is the most common cause of statin intolerance”

## Latin America Guidance

**Fig. 1:**
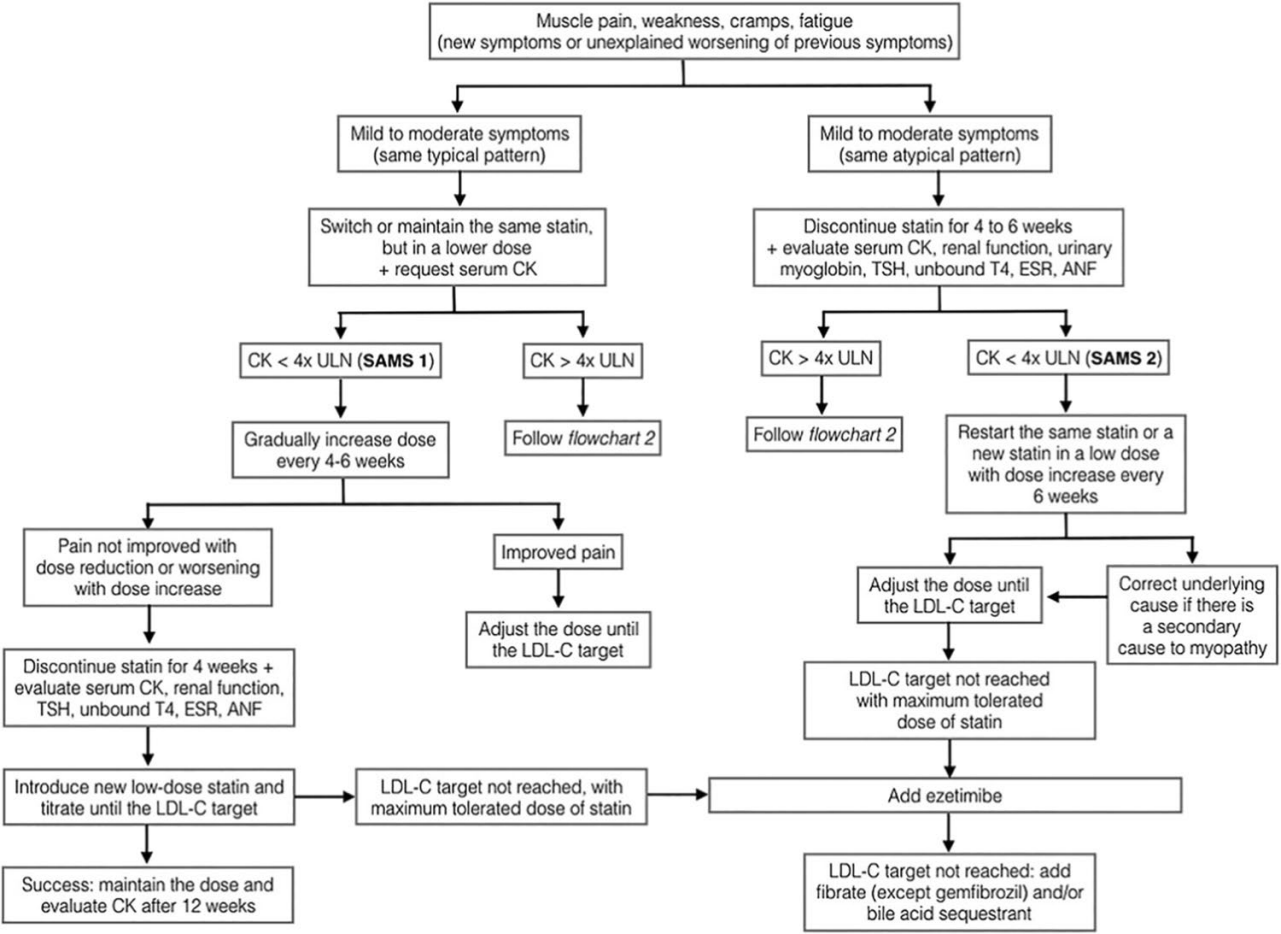
Latin America guidance algorithm for Statin Intolerance (reprinted from: Sposito AC, et al. *Curr Med Res Opin*. 2017; 33(2):239-51, with permission from Taylor and Francis Ltd., http://www.tandfonline.com/) [4•]

In 2016, the Luso-Latin American Consortium (LLAC) released a consensus definition, and guidance on the management of SAMS (Table 1) [4•]. The LLAC definition of statin intolerance includes pharmacologic criteria, symptoms, and etiology. Figure 1 describes the guidance algorithm for statin intolerance.

The guidance reports the seven SAMS standards (SAMS 0 to SAMS 6) ranging from asymptomatic increases in creatinine kinase (CK) < 4× upper limit of normal (ULN) to necrotizing autoimmune myositis [4•]. The LLAC uses an elevation of CK > 7× ULN or any CK value ≥ 1000 IU/L as the cut point for changing clinical management in statin users. LLAC recommends frequent monitoring of CK if statin therapy is altered or halted based on CK elevation and/or intolerable muscle symptoms. For patients with small increases in CK (< 4× ULN) or asymptomatic patients with CK increases between 4 and 7× ULN, a withdrawal period is not necessary, and the same statin may be restarted at a lower dose, or a different statin may be initiated. If CK elevation between 4 and 7× ULN is accompanied by intolerable muscle symptoms, the guidance recommends statin discontinuation for 4-6 weeks before rechallenging with a low-dose statin and careful monitoring of CK. However, regardless of symptom presence, if CK elevations > 7× ULN, the statin should be discontinued for 4-6 weeks, and a new CK measurement should be obtained prior to re-initiating therapy. In cases where CK levels remain elevated after a washout period, patients should be more thoroughly evaluated for secondary causes via thorough clinical and laboratory evaluation. Of note, all treatment strategies that include a discontinuation are followed by a rechallenge, either with the same statin, and dose, or with a different one.

The LLAC endorses that for all patients with CK elevations and/or muscle symptoms, the highest tolerated dose of statins should be kept, and ezetimibe should be the first therapeutic addition. If statin and ezetimibe therapy is insufficient to achieve specific lipid targets, complementary therapy with bile acid sequestrants, fibrates, or phytosterols may be considered. However, the LLAC does not endorse either the use of niacin as add-on lipid-lowering therapy or intermittent statin dosing. The LLAC noted that variability in low-density lipoprotein cholesterol (LDL-C) with intermittent statin dosing was associated with increased cardiovascular mortality and therefore found the recommendation inadequate. This guidance was published prior to the outcome data for proprotein convertase subtilisin/kexin type 9 (PCSK9) monoclonal antibodies.

Patient centeredness has been a central component of managing SAMS, especially when attempting to avoid the “nocebo” effect to prevent negative expectations and to counter existing bias. The LLAC guidance references that previous studies have shown that the “nocebo” effect is attenuated when multiple choices of treatment are made available to the patient, and patients are given the opportunity to choose the option they most prefer. The LLAC was effectively able to detail SAMS, while providing guidance to maximize statin adherence and mitigate the risk of muscle injury.

### European Guidance

**Fig. 2:**
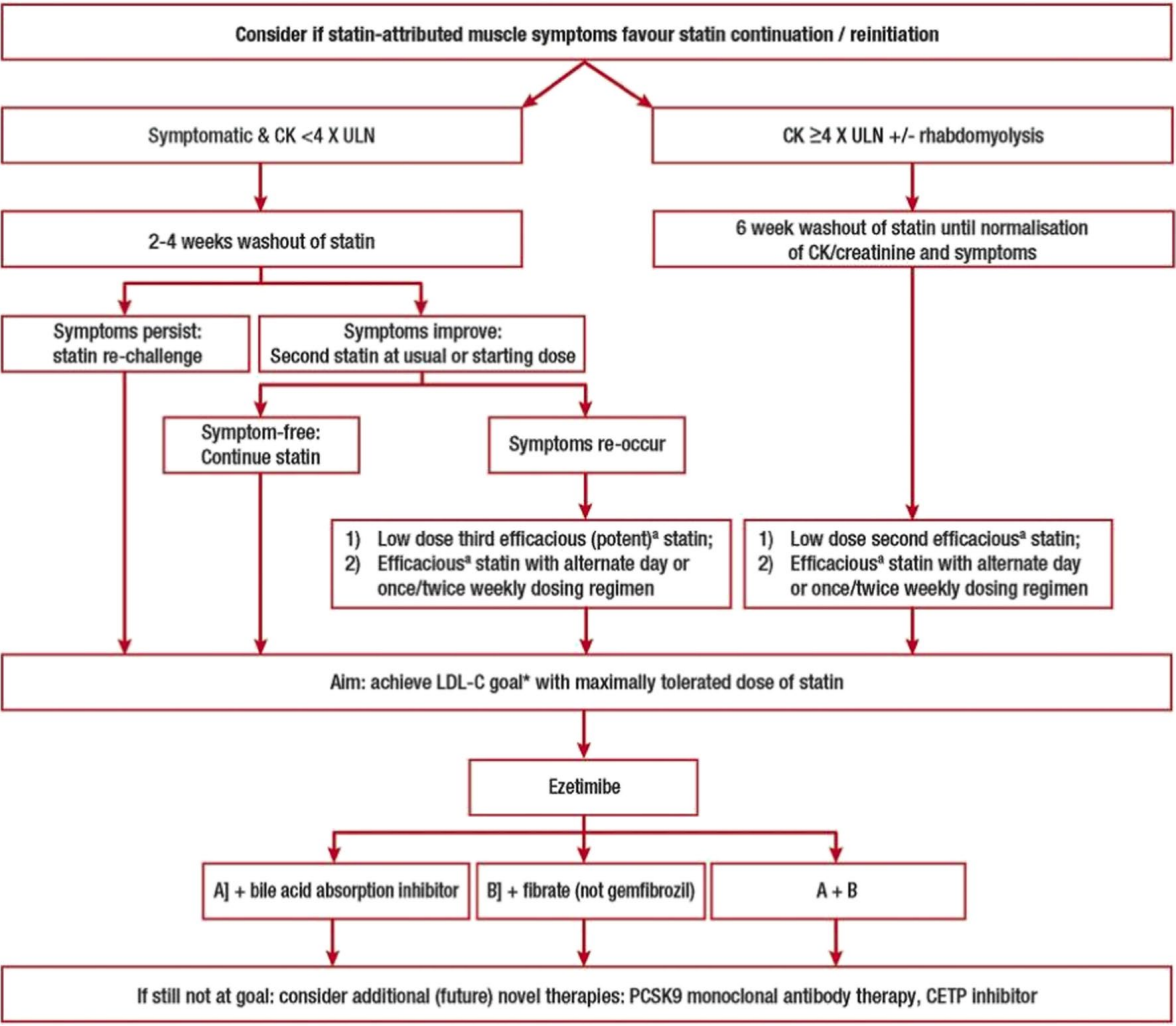
European Guidance algorithm for Statin Intolerance (reproduced from: Stroes et al. *Eur Heart J*. 2015; 36(17):1012-22, by permission of Oxford University Press) [5•]

In 2015, the European Atherosclerosis Society (EAS) Consensus Panel released recommendations for assessing, evaluating the cause of, and managing patients with SAMS (Table 1) [5•]. The EAS proposed a clinical definition that bases the probability of SAMS being caused by statins on the patients’ symptoms, and their temporal relationship with statin initiation, discontinuation (or dechallenge), and repetitive rechallenge [5•]. While the EAS recognized that this clinical definition might not be appropriate for regulatory purposes, they suggest that by focusing more on clinical diagnostic criteria, and providing a structured work-up, individuals with “clinically relevant” SAMS can be offered alternative regimens that will address both the patient’s symptoms and their ASCVD risk [5•]. Figure 2 describes the guidance algorithm for statin intolerance.

The EAS Consensus Panel further differentiates SAMS by CK levels to provide more individualized management [5•]. Most patients that report muscle symptoms have normal or mild/moderately elevated CK levels (<4× ULN), but the differentiation of CK level and ASCVD risk can assist in deciding if statin therapy should be continued [5•]. For example, patients reporting muscle symptoms who have normal or mildly elevated CK levels but only a low ASCVD risk may not warrant statin therapy and instead can receive counseling on lifestyle changes, such as blood pressure control and low-fat diet, whereas for patients with normal or mildly elevated CK levels, and high ASCVD risk, there should be an in-depth discussion of the benefits of continuing statin therapy despite muscle symptoms. It is especially apparent in these cases how the EAS Consensus Panel’s detailed, clinically based, SAMS definition and therapeutic flowchart for management provides helpful tools in clinical practice.

If patients are found to have true SAMS and are not at their low-density lipoprotein cholesterol (LDL-C) goals, then other additional therapies should be employed. The panel suggests a vegetarian diet, ezetimibe, nutraceuticals including psyllium and plant stanols, bile acid sequestrants, and fibrates. This guidance was published prior to the outcome data for proprotein convertase subtilisin/kexin type 9 (PCSK9) monoclonal antibodies. This guidance discusses the possible underlying pathophysiological causes of SAMS. While current research indicates that it is possible statins decrease mitochondrial function, attenuate energy production, and alter muscle protein degradation, the EAS Consensus Panel suggests that further research into the pathophysiology of SAMS could present new therapeutic potential [5•].

In 2017, the EAS Consensus Panel provided an update to their statement on SAMS, where they further stressed the importance of allocating adequate time to managing patients at high risk of cardiovascular events who are also experiencing muscle symptoms [6]. Time is needed to explain the benefits of statin therapy, provide reassurance, and assess statin dechallenge/rechallenge, which is crucial for differentiating true SAMS [6].

### Canadian Guidance

In 2016, the Canadian Consensus Working Group (CCWG) published a comprehensive statin-intolerance definition that integrates a practical concept of “goal-inhibiting statin intolerance” (GISI) (Table 1) [7•]. The CCWG defines statin intolerance as a clinical syndrome marked by the presence of adverse effects after a trial of two different statins; specifically, the CCWG recommends utilizing atorvastatin (10-80 mg) and rosuvastatin (5-40 mg) in initial rounds of therapy. The CCWG explains myalgia, cognitive dysfunction, glycemic control, and gastrointestinal effects among the most common adverse effects of statin therapy [7•].

Specific to SAMS, the CCWG emphasizes the importance of obtaining baseline CK levels prior to statin therapy; discouraging patients from taking supplements to avoid anticipated myalgia symptoms; and appropriately using these data in conjunction with CK metrics according to ethnicity and sex [4•].

**Fig. 3:**
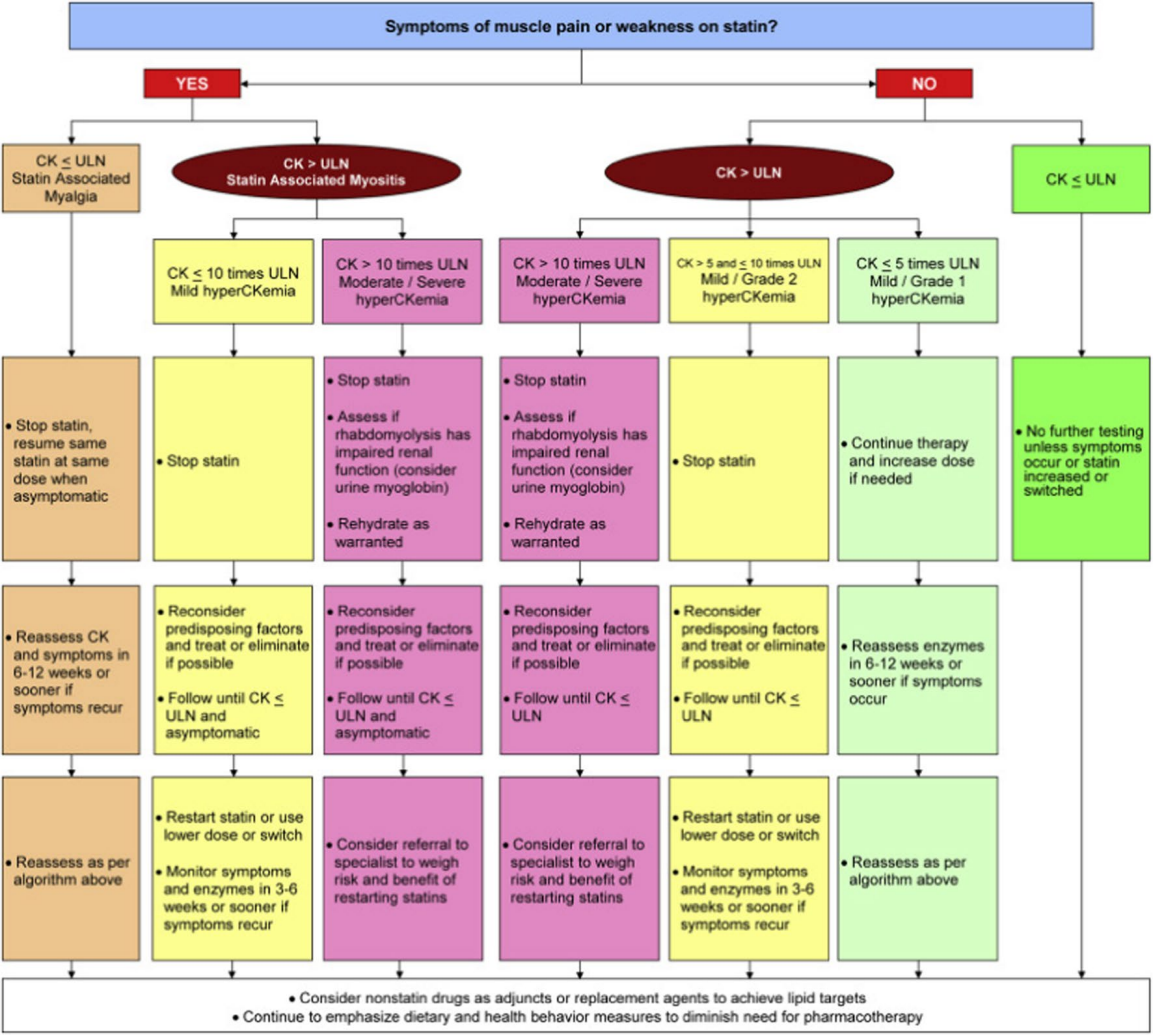
Canadian guidance algorithm for Statin Intolerance (reprinted from: Mancini et al. *Can J Cardiol*. 2016; 32(7 Suppl):S35-65, with permission from Elsevier) [7]

Patients who do not reach their lipid-lowering goals due to discontinuation of statin therapy precipitated by statin-associated adverse effects are designated as GISI. Additionally, the CCWG describes “goal-inhibiting statin resistance” (GISR) as a phenomenon in patients who do not achieve expected benchmarks of lipid lowering through the use of maximally tolerated statin doses. The differentiation of these terms helps explain when a lack of statin efficacy (GISR) prevents achievement of lipid-lowering goals, whereas situations when patients do not reach lipid-lowering benchmarks due to statin-associated adverse effects are defined as GISI. Figure 3 provides the recommended course of action for patients that meet the criteria of CCWG [4•].

Common throughout the CCWG statement is the theme of establishing trust with patients during the entirety of assessment for statin use and subsequent management. The first pillar to goal-inhibiting statin intolerance management is identifying and explaining the strong indications that exist for statin use. Once indication for therapy is established, the group emphasized the importance of understanding risk factors that may predispose patients to experience statin-associated adverse effects and limit statin use, while continually integrating lifestyle modification goals into the overall management of the patient. Non-modifiable risk factors that may predispose patients to statin-associated adverse effects include ages above 80 years old, female sex, family history of myopathies with or without statin therapy, and Asian ethnicity [7•]. Modifiable risk factors that may predispose patients to statin-associated adverse effects include a high statin dose, alcohol and illicit drug use disorders, and several classes of medications [7•]. The CCWG explains that dietary counseling suggests the consumption of phytosterol-containing foods in combination with statin therapy may help patients reach their lipid-lowering goals, while also discouraging patients from taking over the counter supplements to avoid myalgia symptoms. Moreover, the group posits that patients should complete laboratory testing at the initiation of statin therapy and at the first follow-up visit after statin initiation to establish an understanding of a patient’s baseline and response related to statin therapy. Sensible laboratory testing and follow-up helps build trust with patients and is a critical step of goal-inhibiting statin intolerance management.

## United States Guidance

In 2014, the National Lipid Association (NLA) created a definition of statin intolerance that included the concept of “real or perceived,” which at the time, was missing from other definitions [10]. In 2022, the definition was updated further to include a continuum of statin intolerance (Table 1) [8•]. As newer therapies have been added to the armamentarium of lipid therapy, the idea of complete and partial intolerance has become even more clinically relevant. Partially intolerant patients can still tolerate some statin dose, however, due to an adverse effect, not a dose that is high enough to achieve adequate lowering of their atherogenic lipoproteins.

The guidance further described other therapeutic options to decrease atherogenic lipoproteins. As well as described the “nocebo” effect as a possible reason for intolerance, however, according to the writing group, should not be a reason to delay additional therapy, especially in high-risk patients. However, once additional therapy is initiated, finding a tolerable statin regimen should still be a goal of care, as complete statin intolerance is very rare.

**Fig. 4:**
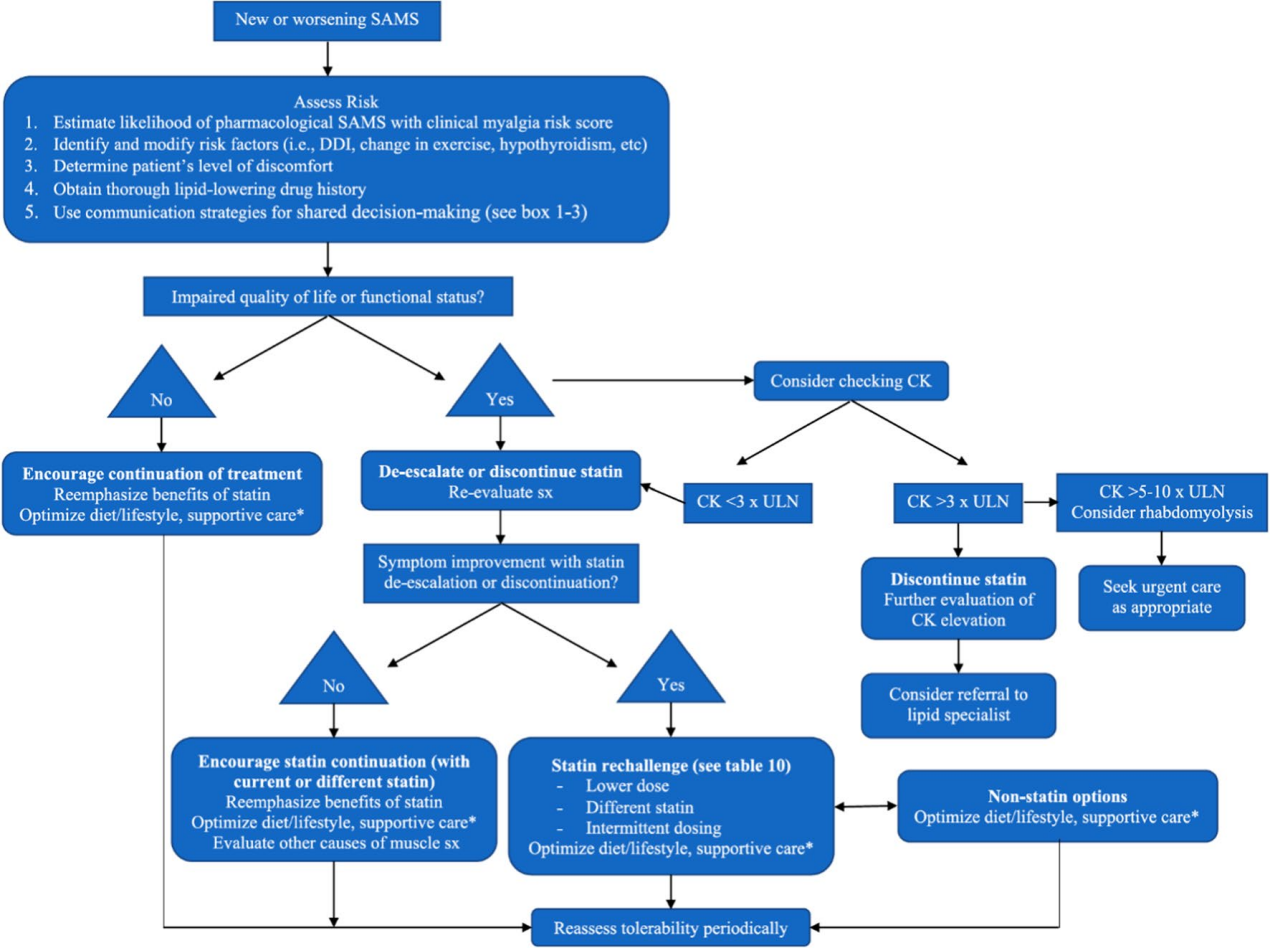
United States guidance algorithm for Statin Intolerance (reprinted from: Warden BA, et al. *J Clin Lipidol*. 2023 Jan-Feb;17(1):19-39, with permission from Elsevier) [9••]

In 2022, the NLA published a guidance on SAMS [9••]. It defines SAMS as “all muscle symptoms temporally related to statin use but without regard to causality” [9••]. The perspective describes patient-centered clinical and communication strategies to mitigate SAMS and provides recommendations to improve statin adherence and patient outcomes [9••]. It recommends four treatment strategies: (1) optimize lifestyle interventions, (2) mitigate risk factors associated with muscle symptoms, (3) alter statin dose to improve tolerability, and (4) use non-statin medications (Fig. 4) [9••].

### Similarities and Differences Among Statin Intolerance Guidance

An overview of the similarities and differences among the statin intolerance guidance documents is available in Table 2. The United States guidance on statin intolerance was the most recently published, while others were almost 5 years old. The year of publication accounted for some of the differences among the guidance documents. The Latin American, European, and Canadian guidance were published prior to the outcome studies for the PCSK9 monoclonal antibodies. Recent publications have shown PCSK9 monoclonal antibodies as an effective addition to lowering LDL-C and reduce ASCVD risk, when added to statin therapy [11, 12]. None of the guidance documents recommends the use of niacin. There are significant differences in the recommendations for the utilization of CK monitoring. All guidance documents provide recommendations for challenge and rechallenge of statin therapy but differ in the intervals and quantity. Both the European and Canadian guidance recommend phytosterols, and all do not recommend the use of coenzyme Q10. All guidance documents but the European mention the nocebo effect.

**Table 2:** Similarities and differences among international statin intolerance guidance

Characteristics	Latin America	Europe	Canada	United States
Publication year	2017	2015/2017	2016	2022
Intermittent dosing	Not recommended	Recommended	Recommended	Recommended
PCSK9 monoclonal antibodies	Discussed potential benefits; awaiting outcomes	Discussed potential benefits; awaiting outcomes	Discussed potential benefits; awaiting outcomes	Recommended, as adjunct to statin therapy, if therapeutic objective not met
Niacin	Not recommended	Not recommended Niacin withdrawn from European market	Not recommended No data to evaluate effectiveness of niacin in statin intolerant patients	Not recommended
Creatine kinase (CK) testing				
Baseline	No comment on baseline CK measurement	Recommend baseline CK testing to subdivide SAMS classifications	Recommend baseline CK testing	Select populations: significant drug interactions, underlying chronic diseases, prior severe statin myopathy
Routine	Recommend routine measurement when introducing new drug or increasing statin dose	Do not recommend regular CK testing with statin therapy due to rarity of symptoms		
Therapy driven	Treatment plan differentiated by CK levels	Only monitor CK levels if CK decreases after stopping statin therapy or if patient is having SAMS and has 4< CK > 10 ULN	Treatment plan differentiated by CK levels	
		Treatment plan differentiated by CK levels		
Symptom based	Recommend CK level in patients with mild to moderate symptoms		Recommend CK testing in statin intolerant patients at time of first follow-up after statin therapy, switching to higher dose, or switching to a different statin	Useful in those with suspected myopathy or rhabdomyolysis
Challenge/rechallenge interval and quantity	Frequency of rechallenge dependent on SAMS classification, between 4 and 6 weeks	Frequency of rechallenge dependent on CK level and symptoms	Decision guided by occurrence and failure of systematic challenge, dechallenge, rechallenge	Rechallenge recommended to meet criteria for statin intolerance
			Rechallenge is not recommended if: significance of symptoms (CK > 5× ULN; ALT >3× ULN); patient refuses to retry same statin at lower dose or frequency	Recommend addition of non-statin therapies in patients who are at very-high risk, while attempting to continue rechallenge with other statin regimens
Nutraceuticals Recommended		Psyllium, plant sterols, portfolio diet	Phytosterols and phytosterol-containing products	
Not recommended	Coenzyme Q10	Red yeast rice, coenzyme Q10	Vitamin D, red rice yeast, coenzyme Q10, berberol, oyster mushrooms	Vitamin D, coenzyme Q10
Insufficient evidence	Vitamin D			
No cardiovascular benefit	Red yeast rice, phytosterols, OTC fish oils			
Nocebo effect	Comments on nocebo effect and strategies for mitigation	No mention	Advise patients of nocebo effect	Nocebo effect influences patient’s experiences and therefore is clinically relevant for a tolerable statin regimen

## Conclusions

The common theme throughout all these guidance documents is the importance of statin therapy to reduce ASCVD and continual adherence to treatment. Because AEs occur and inhibit patients from achieving adequate lowering of their atherogenic lipoproteins, trial and rechallenge of statin therapy, as well as addition of non-statin therapies, especially in high-risk patients, is also undisputed. The main differences stem from laboratory monitoring and the classification of the severity of the adverse effect. A common theme resides among all the guidance documents that most patients can tolerate statins. For those patients who cannot, healthcare teams need to evaluate, rechallenge, educate, and ensure adequate reduction of atherogenic lipoproteins.

## Data Availability

All data generated during and analyzed during this study are included in the published article.
